# Harmful Effects of Microplastics and Nanoplastics in Human Body Systems: A Systematic Review

**DOI:** 10.3390/diseases14030088

**Published:** 2026-02-27

**Authors:** Precious Patrick Edet, Amal K. Mitra, Melissa Dennis, Md S. Zaman

**Affiliations:** 1 William Magee Institute for Student Wellbeing, University of Mississippi, Oxford, MS 38677, USA; pedet@olemiss.edu; 2 Department of Public Health, University of Mississippi, Oxford, MS 38677, USA; 3 Department of Public Health, Julia Jones Matthews School of Population and Public Health, Texas Tech University Health Sciences Center, Abilene, TX 79601, USA; 4 University of Mississippi Libraries, University of Mississippi, Oxford, MS 38677, USA; mdennis@olemiss.edu; 5 Department of Biological Sciences, Alcorn State University, Mississippi, MS 39096, USA; zaman@alcorn.edu; 6 Department of Biology, South Texas College, McAllen, TX 78503, USA

**Keywords:** cardiovascular, gastrointestinal, human health, microplastics, musculoskeletal, nanoplastics, reproductive, respiratory

## Abstract

Background: Microplastics and nanoplastics (MNPs) are ubiquitous environmental contaminants from plastic degradation, leading to human exposure through ingestion, inhalation, and dermal contact. While emerging evidence suggests potential health effects, comprehensive human-specific data remain limited. Objective: To systematically review evidence on MNP exposure and health impacts across human organ systems. Methods: Following PRISMA guidelines, we searched Embase, Environment Complete, MEDLINE, and Scopus for peer-reviewed English-language studies published between 2020 and 2025 that reported MNP exposure in adult human populations and addressed at least one organ system. Thirty studies met inclusion criteria, and all clinical studies were assessed for risk of bias using the Newcastle–Ottawa Scale (NOS) Results: Clinical studies consistently detected MNPs in human blood, thrombi, feces, and respiratory and reproductive tissues. Higher MNP burdens correlated with increased disease severity across cardiovascular, gastrointestinal, respiratory, musculoskeletal, and reproductive systems. In vitro studies using human-derived cell lines demonstrated that MNPs penetrate cells and disrupt cellular processes, inducing oxidative stress, cytotoxicity, mitochondrial dysfunction, inflammation, DNA damage, and apoptosis. Toxic effects were size-, polymer-, and concentration-dependent, with smaller particles exhibiting greater cellular uptake and toxicity. Conclusions: Human MNP exposure is widespread and associated with adverse biological effects across multiple organ systems. Further interdisciplinary research is needed to establish causal relationships and inform risk assessment and regulatory frameworks for plastic-associated contaminants. Other: This research received no external funding. The research protocol was registered with PROSPERO (Registration ID number CRD420261284559).

## 1. Introduction

Plastic production has increased exponentially over the past several decades, exceeding 400 million metric tons annually, with a substantial proportion ultimately fragmenting into microplastics (MPs; <5 mm) and nanoplastics (NPs; <1 µm) through physical, chemical, and biological degradation processes [1,2]. These micro- and nanoplastics (MNPs) consist of a wide range of polymer types, most commonly polyethylene (PE), polypropylene (PP), polystyrene (PS), polyethylene terephthalate (PET), and polyvinyl chloride (PVC), which differ in their chemical composition, density, and resistance to degradation [3,4]. These differences influence how MNPs persist in the environment, interact with biological tissues, and potentially contribute to toxicological effects [3,4]. MNPs are ubiquitous environmental contaminants, detected in air, water, soil, food products, and consumer goods, raising growing concerns about chronic human exposure across the life course [5,6].

Human exposure to MNPs occurs primarily through ingestion, inhalation, and dermal contact [7], with mounting evidence demonstrating their presence in biological matrices such as blood, stool, lung tissue, placenta, semen, and breast milk [6,8,9,10,11,12]. These findings challenge earlier assumptions that plastics are biologically inert and instead suggest that MNPs may translocate across epithelial barriers, enter systemic circulation, and accumulate in human tissues. Importantly, MNPs can do more than cause harm on their own; they can also act as carriers for other hazardous substances. During manufacturing, plastics are often mixed with chemical additives such as phthalates and bisphenols, which are known to interfere with hormonal function, including estrogens, androgens, and thyroid hormones [13,14]. This means that exposure to MNPs could increase the amount and variety of harmful substances reaching the body, potentially worsening inflammation and toxicity beyond the effects of the MNPs alone [15].

Current estimates suggest that individuals may ingest tens of thousands of microplastic particles each year through food, drinking water, and air [16,17]. In a comprehensive exposure assessment, Cox et al. [16] synthesized 402 data points from 26 studies encompassing more than 3600 processed samples to estimate MP intake across approximately 15% of the average American diet. Their findings indicated that annual MP ingestion ranged from approximately 39,000 to 52,000 particles, depending on age and sex. Notably, exposure was substantially higher among individuals who primarily consumed bottled water, with estimated intakes reaching 90,000 MP particles per year, compared with approximately 4000 MP particles per year among those only consuming tap water, highlighting drinking water source as a major determinant of exposure [16]. Inhalation exposure also represents a substantial contributor to overall MNP body burden, particularly in indoor environments where synthetic textiles and household dust are major sources of airborne MP [17,18]. When inhalation is incorporated into exposure estimates, annual MP intake is projected to increase to approximately 74,000–121,000 MP particles per person [16]. Occupational environments, such as plastic manufacturing, recycling facilities, and textile industries, may further elevate exposure levels due to sustained contact with airborne plastic particles, placing workers at higher risk of MNP contamination compared with the general population [19]. These findings underscore MNPs as a population-level environmental exposure with relevance for epidemiologic research and public health surveillance.

Emerging evidence suggests that MNP exposure in human may be relevant across multiple body systems, including the cardiovascular, respiratory, gastrointestinal, immune, and reproductive systems. For example, detection of MNPs in circulating blood, lung tissue, and placental samples raises concern regarding systemic distribution and potential impacts on organ-level function [8,11,12,20]. However, the extent to which MNPs contribute to adverse health outcomes in humans remains limited, in part due to variability in exposure assessment, particle characterization, and outcome measurement across studies. Many reviews have focused on environmental contamination or animal models, while fewer have systematically synthesized evidence specific to adult human populations and human-derived cellular systems. Additionally, variation in exposure assessment methods, outcome measures, and study quality complicates efforts to draw cohesive conclusions regarding biological plausibility and potential health risks of MNPs.

Despite the growing body of research, several critical knowledge gaps persist. First, the majority of human studies rely on cross-sectional designs with small, convenience-based samples, limiting causal inference and generalizability to broader populations. Second, standardized protocols for MNP detection, quantification, and characterization in human biological samples remain lacking, contributing to high variability in reported concentrations and making cross-study comparisons challenging. Third, while in vitro studies have elucidated potential mechanisms of cellular toxicity, the translation of these findings to organ-level dysfunction and clinical disease outcomes in humans remains poorly understood. Fourth, dose–response relationships between MNP exposure levels and health outcomes have not been systematically established, particularly at environmentally relevant concentrations. Fifth, longitudinal studies examining the cumulative effects of chronic MNP exposure across the life course are virtually absent from the literature. Finally, there is limited understanding of how individual factors such as age, sex, genetic susceptibility, pre-existing health conditions, and co-exposures to other environmental contaminants may modify the health effects of MNPs. Addressing these gaps is essential for establishing the public health significance of MNP exposure and for developing evidence-based risk assessment frameworks.

A comprehensive synthesis that integrates both clinical and in vitro evidence is therefore needed to better characterize how MNP exposure may influence human body systems and to identify key gaps for future research. Accordingly, this systematic review aims to synthesize recent evidence (2020–2025) on the health impacts of MNPs across human body systems. By integrating findings from clinical studies involving adult human participants and in vitro studies using human-derived cell lines, this review provides an updated and interdisciplinary assessment of MNP exposure and its potential implications for various human body systems.

This synthesis has important implications for multiple stakeholders. For researchers, it provides a foundation for designing mechanistic and epidemiological studies that address existing knowledge gaps and advance understanding of MNP toxicology. For public health professionals, the findings highlight the need for exposure reduction strategies, particularly among highly exposed populations such as occupational workers and consumers of bottled water. For policymakers and regulatory agencies, this review underscores the urgency of developing standardized methods for MNP monitoring in environmental and biological samples, establishing exposure limits, and implementing preventive measures to reduce plastic pollution at the source. For healthcare providers, awareness of MNP-associated health risks may inform clinical screening and patient counseling, particularly for vulnerable populations such as pregnant women and individuals with pre-existing cardiovascular, respiratory, or gastrointestinal conditions. Understanding these relationships is essential for informing future epidemiologic research, risk assessment efforts, and evidence-based public health strategies addressing plastic-related exposures. Findings are also essential to guide exposure monitoring efforts and inform regulatory decision-making related to plastic-associated contaminants. Ultimately, this review aims to catalyze interdisciplinary action to mitigate the health impacts of one of the most pervasive environmental contaminants of the 21st century.

## 2. Materials and Methods

### 2.1. Protocol

The Preferred Reporting Items for Systematic Reviews and Meta-Analysis (PRISMA) guidelines (2009) were used as a guide to record the review process [21]. This systematic review encompassed various stages: defining keywords, database searches for article selection, quality appraisal of selected studies, data extraction and analysis, and presenting and interpreting results. The research protocol was registered with PROSPERO (Registration ID number CRD420261284559).

### 2.2. Eligibility Criteria

Studies were eligible for inclusion if they were published between 2020 and 2025, written in English, and conducted in any geographic location. Eligible studies included adult human populations (≥18 years), with no restrictions on sex or gender. Both original research articles and review articles (including systematic reviews and meta-analyses) were considered. To meet inclusion criteria, studies were required to report on microplastic and/or nanoplastic (MNP) exposure in humans and address at least one human organ or body system. This included studies examining (1) the prevalence or detection of MNPs in human tissues or biological samples, and/or (2) the biological mechanisms or pathways through which MNPs exposure impacts human organ systems.

Studies were excluded if they were published before 2020 or were not peer-reviewed full-text articles. Reports, letters to the editor, commentaries, conference abstracts, preprints, and the gray literature were excluded from this review. The inclusion and exclusion criteria for the review are provided in Table 1.

### 2.3. Search Strategy and Information Sources

The study includes four databases with broad subject coverage in order to identify articles from a variety of disciplines: Embase, Environment Complete, MEDLINE, and Scopus. The research strategy combined at least one word or phrase from each area of focus from the research questions. Several search strategies were performed between March and July 2025, adjusting search terms for each database until agreeing on a final search that best accommodated each database’s specific filtering systems. The final search included the following terms: (nanoplastics OR microplastics) AND (organ OR health OR system OR genetic) AND (mechanism OR complication OR contamination OR toxic) and limited the date to the previous 5 years from 2020 to 2025 (Table 2). The inclusion criteria to screen studies included focusing only on peer-reviewed, academic journal article studies with adult participants (aged 18 or older) written in English.

### 2.4. Screening Guidelines

Articles identified for review were exported to Zotero reference management software (version 1.0.2), with duplicates removed. Remaining articles were imported into Rayyan for initial screening by title and abstract according to their relevance to the research question. At least two reviewers screened each abstract independently to determine eligibility using the identified inclusion and exclusion criteria. Conflicts between reviewers were resolved through discussion using the research questions to guide inclusion criteria and noting reasons for excluding articles. Upon completion of the initial screening process, 271 abstracts were selected for full review.

**Figure 1:**
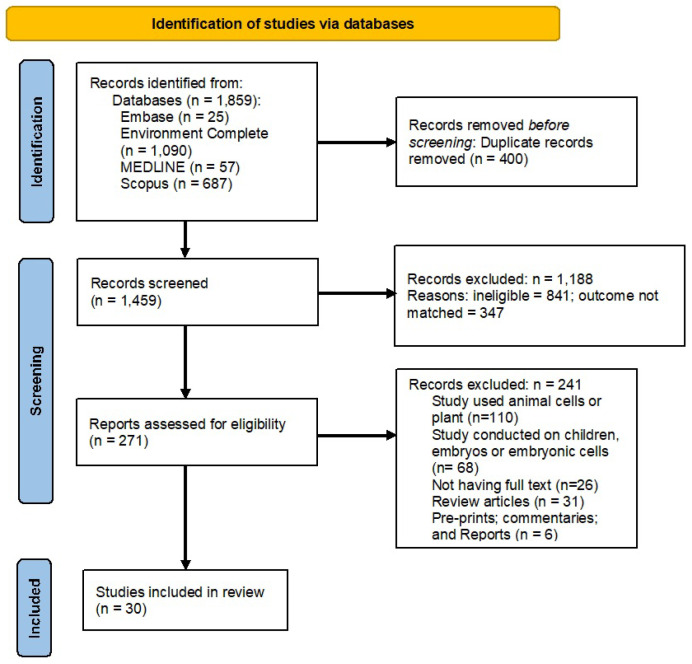
PRISMA flow diagram for the articles retrieved, screened, and included in the review.

During the second phase of screening, full-text articles of eligible abstracts (*n* = 271) were retrieved and reviewed independently by at least two researchers on whether they answered the research questions and fulfilled the inclusion criteria. A cross-reference list of selected review articles was also examined to identify additional potentially eligible studies. Conflicts were managed by group discussions and studies were included if consensus was reached by all researchers. A total of 30 articles were included in the systematic review, after the full-text review process was completed and we subdivided into clinical and in vitro studies based on the study design. The PRISMA flow chart (Figure 1) exhibits the search and inclusion process for the systematic review.

### 2.5. Quality Assessment of Clinical Studies

#### 2.5.1. Quality Appraisal

Clinical studies were appraised for quality in Microsoft Excel. Standards for the critical appraisal and the rating scale of the 13 clinical studies were pre-defined prior to appraisal of articles. The critical appraisal tool was adapted from Joanna Briggs Institute [22], and study quality was evaluated across the following six domains: sample size, study design, sampling method, use of standardized data instruments, data validation, and data analysis. Sample size was scored on a four-point scale based on quartile distribution (≤30 = 1 point; 31–45 = 2 points; 46–91 = 3 points; >91 = 4 points). Study design was scored from 0 to 5 points, with higher scores assigned to more rigorous designs (literature reviews = 0; systematic reviews = 1; cross-sectional studies = 2; case–control studies = 3; cohort studies = 4; clinical trials and meta-analyses = 5). Sampling method was scored as 0 for convenience sampling and 1 for random sampling. The use of standardized data collection instruments (e.g., validated questionnaires or diagnostic tools) was scored as 1 point if present and 0 if absent. Data validation procedures were similarly scored as 1 point if reported and 0 if not reported. Finally, data analysis methods were scored from 0 to 3 points, with points assigned based on analytical rigor (none = 0; descriptive statistics = 1; univariate or bivariate inferential analyses = 2; and multivariate or multiple analytical methods = 3). The sum of these domains yielded a total quality appraisal score ranging from 0 to 15, with higher scores indicating greater methodological rigor.

Based on the above-mentioned criteria, the three researchers (PPE, AKM, and MSZ) rated each of the 13 clinical studies independently from a range of 1 to 15. Due to having no major inter-observer variations in the evaluation of the quality of the studies, an average of the three scores was presented.

#### 2.5.2. Risk of Bias Assessment

The risk of bias of the included clinical studies (*n* = 13) was assessed using study design-specific, validated tools. Case–control and cohort studies were evaluated using the Newcastle–Ottawa Scale (NOS) (see Appendix A) [23], which assesses risk of bias across the following three domains: selection of study groups, comparability of groups, and ascertainment of exposure or outcomes. Studies were awarded up to nine stars, with higher scores indicating lower risk of bias. Cross-sectional studies were assessed using the adaptation of the NOS for cross-sectional studies (association-studies) (NOS-xs) (see Appendix B) [24], which assesses risk of bias across three domains: study sample selection, assessment of exposure and outcome, and confounding factors. Studies were awarded up to nine stars, with higher overall scores indicating lower risk of bias.

Each study was independently assessed by two reviewers (PPE and AKM), and discrepancies were resolved through discussion and consensus. Overall risk of bias scores for both the NOS and NOS-xs were categorized as either high risk (0–3), moderate (4–6), or low risk (7–9) [24,25].

### 2.6. Data Extraction

A structured data extraction template was created to systematically capture key information from each included study. Extracted variables included study design, year of publication, country where the study was conducted, primary findings, and the human body systems affected (Table 3 and Table 4). A thematic analysis approach was employed to systematically identify, examine, and summarize patterns and themes emerging across the studies [26,27]. Authors (PPE, AKM, and MD) independently reviewed the extracted data to identify recurring themes, after which the research team met to discuss and agreed upon the final themes used for synthesizing the results.

## 3. Results

A summary of the methodology, key findings, impacts of MNPs on human body systems, quality appraisal, and the countries of study are presented in Table 3 and Table 4. Of the 30 included studies, 17 were conducted in China, two in Taiwan, two in Italy, and one each in Germany, India, Poland, South Korea, Spain, Switzerland, Tunisia, Turkey, and the United States. Thirteen of the studies were clinical in nature, employing either quantitative or mixed methods designs to assess the associations between MNP exposure and health outcomes in human participants, while the remaining 17 were in vitro studies exploring cellular and molecular mechanisms of MNP toxicity across multiple human body systems. These in vitro investigations primarily used human cell lines, including cardiovascular, gastrointestinal, immune, lymphatic, reproductive, and reproductive cells, to examine endpoints such as oxidative stress, mitochondrial dysfunction, apoptosis, inflammation, cytokine release, and autophagy disruption. Collectively, in vitro studies provided mechanistic insights into how MNPs can penetrate cells and tissues and trigger cytotoxic and immunotoxic effects, complementing the findings from clinical research.

Furthermore, all 13 clinical studies utilized various designs, including cross-sectional, cohort, and case–control approaches, often employing convenience sampling to recruit participants visiting healthcare facilities for medical services. These studies were conducted from February 2022 to December 2024, with eight of the 13 studies not reporting the exact dates of data collection. The total sample sizes ranged from 10 to 113, with a median of 45 (1st quartile = 30; 3rd quartile = 91); 6 out of 13 (46%) had sample sizes greater than 45. In terms of standardized tools, only 11 studies utilized standardized tools. Quality appraisal scores ranged from 0 to 15 points. Five studies (38%) scored 11–15 points and were rated as ‘excellent,’ eight studies (62%) scored 6–10 points and were rated as ‘moderate,’ and no studies scored 0–5 points or were rated as ‘poor.’ All studies highlighted a negative impact of MNPs on human body systems.

### 3.1. Impact of MNPs of Human Body Systems: Findings from Clinical Studies

Clinical studies examined the associations between MNPs exposure and health outcomes across multiple human body systems. Studies employed various study designs, including cross-sectional, cohort, and case–control designs.

#### 3.1.1. Cardiovascular System

Wang et al. [28] employed a mixed-methods study to identify the presence of MP in human arterial and venous thrombi, as well as examine the association between MP levels and disease severity. Findings revealed MPs in 80% of thrombi from patients with ischemic stroke, myocardial infarction, or deep vein thrombosis, with the highest median concentrations observed in myocardial infarction cases (141.80 μg/g). Higher MP concentrations were associated with increased disease severity (adjusted β = 7.72, 95% CI: 2.01–13.43). In addition, D-dimer levels were significantly higher in the MP-detected group than the MP-undetected group (8.3 ± 1.5 μg/L vs. 6.6 ± 0.5 μg/L, *p* < 0.001), with laser direct infrared (LDIR) analysis showing polyethylene as the dominant polymer.

Similarly, Yang et al. [29] found significantly higher MP concentrations in patients with acute coronary syndrome compared with controls, with greater loads among patients with acute myocardial infarction than those with unstable angina. Additionally, patients with intermediate to high coronary artery disease risk exhibited significantly greater MP accumulation compared with those at low risk. Increased MP levels were also associated with elevated inflammatory cytokines (IL-6 and IL-12p70) and higher B lymphocyte and natural killer cell counts.

Yu et al. [30] examined blood MP burdens in patients with extracranial artery stenosis (ECAS) and their association with disease severity. Using Py-GC/MS, LDIR spectroscopy, and SEM, MPs were detected in all blood samples, with significantly higher concentrations observed in ECAS patients compared with controls (174.89 ± 24.95 vs. 79.82 ± 31.73 μg/g, *p* < 0.001). Polyvinyl chloride (PVC) and polyamide 66 (PA66) were the most prevalent polymers identified. Importantly, MP concentrations increased with the severity of arterial stenosis, suggesting a dose–response relationship. Elevated MP levels were also associated with prothrombotic alterations, including higher D-dimer levels and prolonged thrombin time, indicating potential effects on coagulation pathways.

#### 3.1.2. Gastrointestinal System

Yan et al. [31] reported significantly higher fecal MP concentrations among patients diagnosed with inflammatory bowel disease (IBD) compared with healthy controls (41.8 vs. 28.0 items/g dry mass). In total, 15 types of MP were detected in feces, with PET (22.3–34.0%) and polyamide (8.9–12.4%) being the most prevalent. MP concentrations were positively correlated with IBD severity, suggesting greater accumulation with worsening disease.

Similarly, Cetin et al. [32] investigated the presence and characteristics of MPs in tumoral and non-tumoral colon tissues from patients diagnosed with colorectal adenocarcinoma and colon tissues from cancer-free controls. Findings revealed increased MP levels in tumoral colon tissues compared with non-tumoral tissues and colon tissues from cancer-free controls. MPs detected ranged in size from 1 to 1299 µm and were primarily composed of polyethylene, poly(methyl methacrylate), and polyamide. Higher MPs level in tumoral tissues suggests a potential association between MP exposure and colorectal cancer.

Wu et al. [33] investigated MP accumulation in fibrotic intestines and adjacent mesenteric adipose tissue of Crohn’s disease patients. Their findings indicated a positive correlation between MP concentrations and the severity of intestinal fibrosis. LDIR spectroscopy revealed that nearly one-third (31.96%) of MPs were 20–50 μm in size. High-risk practices, such as frequent invasive gastrointestinal procedures, were associated with greater MP accumulation. MPs were found at higher concentrations at lesion sites compared with surrounding tissues.

#### 3.1.3. Musculoskeletal System

Li et al. [34] detected MPs in nearly all blood samples, with PVC and PET being the most common polymers. Exposure risk to MPs included sources such as bottled water and take-out containers. MP exposure was linked to toxic effects on osteoblasts and abnormal gene expression, suggesting a potential correlation with osteoporosis progression.

#### 3.1.4. Respiratory System

Lui et al. [35] examined exposure to MPs among patients with community-acquired pneumonia (CAP), including severe CAP (SCAP) and non-severe CAP (NSCAP). MPs were detected in 98% of sputum samples and 94% of bronchoalveolar lavage fluid samples, with mean concentrations of 23.24 and 4.49 μg/g dry weight, respectively. Higher MP exposure was significantly associated with increased risk of SCAP and correlated with alterations in respiratory microbiota, including reduced α-diversity and changes in inflammatory factors.

#### 3.1.5. Reproductive System

Zhang et al. [36] reported a significant association between MP exposure, particularly polytetrafluoroethylene (PTFE), and reduced semen quality. MPs were detected in all semen and urine samples, with participants typically exposed to three to five different polymer types, most commonly polystyrene, polypropylene, and polyethylene. PTFE exposure was associated with lower total sperm count, sperm concentration, and progressive motility compared with unexposed individuals, with additional declines observed as the number of microplastic types increased.

Kim et al. [37] study, which enrolled 13 women aged 25–52, revealed that smaller polystyrene MNPs (100 nm and 1 μm) exhibited higher cellular uptake in human endometrial stromal cells than larger particles, leading to morphological changes, reduced proliferation, and apoptosis at concentrations above 100 μg/mL.

Two studies by Xu et al. [38,39] examined two gynecologic conditions that may be associated with MP exposure: cervical cancer and uterine fibroid. In cervical cancer tissues, exposure to MPs increased with disease progression, with polyethylene (26.73%) and polypropylene (19.80%) being most prevalent types of MP [38]. Metabolomic analyses revealed significant enrichment of amino sugar and nucleotide sugar metabolism pathways, potentially acting as pathways through which MPs may contribute to cervical cancer [38].

Xu et al. [39] further demonstrated that microplastic levels were significantly higher in uterine fibroid tissues compared with normal tissues and tissues from healthy controls (*p* < 0.01). Among the detected polymers, polyethylene exposure was associated with an increased risk of uterine fibroids and showed a positive correlation with fibroid size, suggesting a potential role in fibroid growth and progression. Metabolomic analyses revealed significant alterations in metabolic profiles associated with MP exposure, with enrichment of amino sugar and nucleotide sugar metabolism pathways showing an upward trend, while pathways related to cofactor biosynthesis and platelet activation were downregulated.

Zhang et al. [40] reported that maternal exposure to MP was significantly associated with pregnancy-induced hypertension (PIH). Umbilical cord analyses revealed significantly higher concentrations of polyethylene and polycarbonate in PIH patients compared with controls, with total MP levels 1.46 times higher in PIH cases. Questionnaire data indicated that exposures related to plastic containers and takeout meals were positively correlated with MP levels, while factors such as plastic tableware, seafood consumption, and plastic-packaged beverages were identified as potential but not independent risk factors in multivariate analyses. Importantly, elevated MP exposure was associated with adverse neonatal outcomes, including increased neonatal mortality and lower Apgar scores.

**Figure 2:**
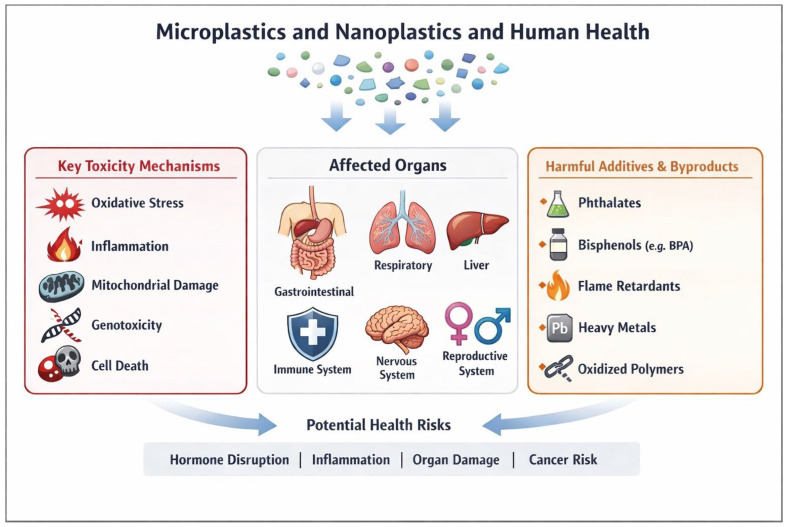
A schematic diagram showing key mechanisms of toxic effects, harmful additives and byproducts of MNPs, potential health risks, and multiple organ systems in human body affected by MNPs.

Figure 2 summarizes the key toxicity mechanisms, harmful additives and byproducts, potential health risks, and multiple human body systems affected by MNPs.

Table 3 summarizes clinical studies included in this review, including study characteristics, major findings, quality appraisal, and risk of bias assessment.

### 3.2. Impact of MNPs of Human Body Systems: Findings from In Vitro Studies

A total of 17 in vitro studies examined the cellular and molecular impact of MNPs exposure across multiple human body systems.

#### 3.2.1. Cardiovascular System

Cardiovascular toxicity was reported across multiple in vitro studies. Ma et al. [41] demonstrated that chronic exposure to polystyrene MNPs impaired viability, contractility, and calcium handling in human iPSC-derived cardiomyocytes, even at low doses. These effects were associated with mitochondrial dysfunction, increased mitochondrial reactive oxygen species (ROS), and exacerbation of hypertrophic phenotypes.

Persiani et al. [42] showed that polyethylene microplastics (PE-MP) and polystyrene microplastics (PS-MP) induced pathological activation of vascular smooth muscle cells, including apoptosis, inflammation, altered migration, and upregulation of cardiovascular disease markers such as RUNX-2 and galectin-3. Similarly, Xue et al. [43] found that PS-MPs activated endoplasmic reticulum (ER) stress pathways and inhibited autophagic flux in AC16 cardiomyocytes, contributing to cellular injury, which was partially reversed by autophagy activation.

#### 3.2.2. Gastrointestinal System

Chen et al. [44] reported that 0.1 and 1 μm PS-MPs entered normal human liver (THLE-2) cells without inducing acute cytotoxicity after 48 h, suggesting potential sublethal or long-term effects. However, metabolomic analyses showed that 90 days of exposure to PS-MPs at an environmentally relevant concentration (0.2 μg/mL) significantly altered cellular metabolic profiles, with particularly pronounced effects observed for nanosized MPs. Guanglin and Shuqin [45] demonstrated that PS-NPs induced inflammatory responses and cell death in esophageal cell lines by promoting iron overload, mitochondrial ROS accumulation, and suppression of mitochondrial autophagy.

Using an in vitro colon model, Nissen et al. [46] reported that combined PE-MP and PS-MP disrupted gut microbial ecology, promoting opportunistic bacterial overgrowth (*Enterobacteriaceae*, *Desulfovibrio* spp., *Clostridium* group I and *Atopobium*–*Collinsella* group) while reducing beneficial taxa, with a sole exception of Lactobacillales. Najahi et al. [47] showed that exposure to PE-MPs and polyethylene terephthalate (PET) MPs of varying sizes (1 µm and 2.6 µm) for 72 h significantly reduced viability of Caco-2 intestinal epithelial cells, increased ROS production, and triggered apoptosis and autophagy through increase in Bax/Bcl-2 signaling and LC3-II, activation of caspase-3, and decrease in p62 expression.

#### 3.2.3. Immune and Lymphatic Systems

Çobanoğlu et al. [48] reported increased genomic instability in human peripheral blood lymphocytes following MP exposure. Specifically, lymphocytes exposed to five different MP concentrations for 48 h exhibited marked increases in micronuclei, nucleoplasmic bridges (NPB) formation, and nuclear buds (NBUD) formation, all well-established biomarkers of chromosomal damage and genotoxic stress. Notably, these genotoxic effects were observed even at lower exposure concentrations, indicating a high sensitivity of lymphocytes to microplastic-induced DNA damage. In contrast, the cytokinesis-block proliferation index (CBPI) remained unchanged across exposure groups, suggesting that MP did not impair cell proliferation or induce cytostasis.

Weber et al. [49] investigated the immunological effects of nanoplastics by exposing human monocytes and monocyte-derived dendritic cells in vitro to particles varying in shape (irregular vs. spherical), size (50–310 nm and polydisperse mixtures), and polymer composition (polystyrene, polymethyl methacrylate, and polyvinyl chloride) at concentrations ranging from 30 to 300 particles per cell. Findings showed that nanoplastic exposure induced cytokine secretion in a concentration-dependent manner, with irregularly shaped PVC nanoplastics eliciting the strongest cytokine release among all tested materials and irregular polystyrene particles triggering significantly greater pro-inflammatory cytokine production compared with their spherical counterparts.

Koner et al. [50] reported that PS-NP exposure resulted in a significant, dose-dependent reduction in macrophage viability, with the highest concentration (500 µg/mL) also leading to suppressed cell proliferation. At elevated exposure levels, PS-NPs induced oxidative stress, accompanied by a marked decrease in mitochondrial membrane potential, indicating mitochondrial dysfunction. In addition, PS-NP exposure caused measurable DNA damage in macrophages, suggesting genomic instability, highlighting size- and dose-dependent immunotoxic effects.

#### 3.2.4. Reproductive System

Zhang et al. [51] demonstrated that PS-NPs entered human ovarian granulosa (KGN) cells and induced dose-dependent increases in cytoplasmic vacuoles, lysosome accumulation, and lipid droplet formation, despite minimal changes in cytotoxicity, calcein intensity, and mitochondrial activity.

#### 3.2.5. Respiratory System

Dong et al. [52] demonstrated that PS-MPs induced cytotoxicity and inflammatory responses in BEAS-2B bronchial epithelial cells through ROS formation, reduced transepithelial electrical resistance, and depletion of tight junction proteins. Decreased α1-antitrypsin expression further suggested increased susceptibility to chronic obstructive pulmonary disease.

Similarly, Halimu et al. [53] reported that PS-NPs promoted epithelial-to-mesenchymal transition in A549 lung epithelial cells, which preludes lung fibrosis. This is accompanied by increased migration, ROS production, NADPH oxidase production, mitochondrial dysfunction, and activation of endoplasmic reticulum (ER) stress pathways, with smaller, positively charged PS-NPs exhibiting stronger effects. Annangi et al. [54] observed significant cellular uptake of polyethylene terephthalate nanoplastics (PET-NPLs) in human primary nasal epithelial cells, resulting in increased intracellular ROS, loss of mitochondrial membrane potential, and activation of the autophagy pathway. PET-NPLs exposure also significantly increased LC3-II protein and p62 expression levels.

Additional mechanistic insights were provided by Han et al. [55], who identified integrin α5β1-mediated endocytosis as a key pathway facilitating PS-NP internalization into lung epithelial cells, leading to mitochondrial Ca^2+^ dysfunction and depolarization, increased ROS production, oxidative damage, inflammation, DNA damage, and necrosis, contributing to lung disease. Winiarska et al. [56] further demonstrated that PS-NPs of sizes 25 or 50nm for 1 or 24 h penetrated bronchial smooth muscle cells and small airway epithelial cells. Exposure to PS-NPs in both cell types resulted in compromised bioenergetic function and mitochondrial dysfunction compared with cells not treated with NP, as evidenced by changes in oxygen consumption rate, extracellular acidification rate. Authors suggest that NPs pose a serious threat to oxidative and glycolytic metabolism.

#### 3.2.6. Vascular System

Chen et al. [57] demonstrated that exposure to PS-MPs at physiologically relevant blood concentrations induced oxidative stress and apoptotic cytotoxicity in human vascular endothelial (EA.hy926) cells by downregulating antioxidant defenses and heat shock proteins. PS-MP exposure was also associated with reduced expression of ROCK-1 and NF-κB, leading to suppression of NLRP3-related inflammatory signaling, and caused vascular barrier dysfunction through depletion of the tight-junction protein zonula occludens-1. However, despite alterations in nuclear receptor NR4A1 expression, PS-MPs did not increase LOX-1 expression, suggesting limited potential to promote atherosclerotic processes under the conditions studied.

Table 4, described below, summarizes in vitro studies included in this review, including study characteristics and major findings.

#### 3.2.7. Certified Reference Materials (CRMs)

Table 5 depicts information on MNP detection and characterization methods, and the use of typical detection limits, polymer specificity, and known biases/limitations for cross-study comparability. However, despite rapid progress in analytical methods, results from studies reviewed were difficult to compare, primarily because of the lack of data and other commonly identified issues such as: (1) laboratories used different sampling, pre-treatment, and analytical procedures rather than harmonized protocols; and (2) results were often not directly comparable in terms of size ranges, units (counts vs. mass), or polymer categories. The use of standardized sampling, preparation, and detection protocols would greatly improve comparability across studies.

In addition, there is a lack of well-characterized plastic particle standards to reflect polymer types and relevant sizes. Therefore, developing and using CRMs is essential for harmonized, comparable MNP measurements across laboratories. CRMs help calibrate instruments, validate analytical methods, and improve quantification accuracy across laboratories.

## 4. Discussion

This systematic review synthesized evidence from 30 studies published between 2020 and 2025 to examine the health impacts of microplastics and nanoplastics (MNPs) across human body systems. The findings reveal that MNP exposure is widespread in human populations and biologically consequential, with detection of MNPs in diverse human tissues and biological fluids, associations between higher MNP burdens and increased disease severity in clinical populations, and demonstration of cellular and molecular mechanisms of toxicity in human-derived cell lines. Collectively, the evidence challenges the long-held assumption that plastics are biologically inert and instead positions MNPs as a potentially significant environmental health threat warranting further investigation and regulatory attention.

### 4.1. Principal Findings and Integration with the Existing Literature

The clinical studies included in this review consistently detected MNPs in human biological samples, including blood, thrombi, feces, sputum, bronchoalveolar lavage fluid, semen, urine, and reproductive tissues. This widespread detection aligns with and extends earlier reports of MNPs in human placenta [20], blood [12], and stool [11], providing further evidence that MNPs can cross physiological barriers and achieve systemic distribution. Importantly, the present review identified associations between elevated MNP concentrations and adverse health outcomes across multiple organ systems, including increased severity of ischemic stroke, myocardial infarction, and deep vein thrombosis in cardiovascular tissues [28]; worsened inflammatory bowel disease and intestinal fibrosis in gastrointestinal tissues [31,33]; and reduced semen quality and associations with gynecological conditions such as cervical cancer, uterine fibroids, and pregnancy-induced hypertension in reproductive tissues [38,39,40]. These findings suggest that MNPs may not simply accumulate passively in tissues but may actively contribute to disease pathogenesis.

The in vitro studies complement these clinical observations by elucidating potential mechanisms through which MNPs exert toxic effects at the cellular level. Consistent across studies was the demonstration that MNPs can be internalized by human cells, disrupt critical cellular processes, and trigger pathological responses including oxidative stress, mitochondrial dysfunction, endoplasmic reticulum stress, inflammation, apoptosis, and autophagy dysregulation [43,47,49,52,53,57]. These mechanisms are biologically plausible contributors to the clinical outcomes observed in human studies and are consistent with toxicological frameworks established for other particulate pollutants, such as fine particulate matter (PM2.5), which similarly induces oxidative stress and inflammation leading to cardiovascular and respiratory disease [58]. The finding that MNP toxicity was generally size-, polymer-, and concentration-dependent further supports a dose–response relationship, with smaller particles (nanoplastics) and certain polymer types (e.g., polystyrene, polyvinyl chloride) exhibiting greater cellular uptake and cytotoxic potential. This is consistent with nanoparticle toxicology principles, wherein smaller particles possess higher surface area-to-volume ratios and enhanced capacity to penetrate cellular membranes and organelles [59].

The present findings also corroborate and extend recent narrative reviews and commentaries on MNP health effects [6,8], which have highlighted the ubiquity of MNP exposure and called for greater research attention. However, this systematic review advances the field by providing a structured synthesis of both clinical and mechanistic evidence published within a recent, well-defined timeframe, thereby offering a more comprehensive and up-to-date assessment of MNP impacts on human body systems.

### 4.2. Potential Mechanisms Linking MNPs to Human Health Outcomes

Based on the in vitro evidence synthesized in this review, several interconnected mechanisms may explain how MNP exposure leads to adverse health effects in humans. First, oxidative stress induced by MNPs appears to be a central pathway of toxicity. Multiple studies demonstrated that MNPs increase ROS production and deplete antioxidant defenses, leading to oxidative damage to lipids, proteins, and DNA [41,50,52,53,54]. This oxidative stress can activate inflammatory signaling pathways, including nuclear factor-kappa B (NF-κB) and NLRP3 inflammasome, resulting in elevated pro-inflammatory cytokine secretion (IL-6, IL-12p70, TNF-α) observed both in vitro and in clinical samples [29,56]. Chronic inflammation is a well-established contributor to atherosclerosis, thrombosis, cancer, and other chronic diseases, providing a plausible link between MNP exposure and the cardiovascular, gastrointestinal, and reproductive pathologies documented in clinical studies.

Second, mitochondrial dysfunction emerged as a recurrent theme across studies. MNPs were shown to disrupt mitochondrial membrane potential, impair oxidative phosphorylation, reduce ATP production, and increase mitochondrial ROS generation [41,52,53,54]. Given that mitochondria are essential for cellular energy metabolism and play critical roles in apoptosis regulation and calcium homeostasis, mitochondrial dysfunction induced by MNPs could contribute to impaired organ function, particularly in metabolically active tissues such as the heart, liver, and kidneys.

Third, disruption of cellular membrane integrity and barrier function was evident in studies examining epithelial and endothelial cells. For example, MNPs reduced transepithelial electrical resistance and depleted tight junction proteins such as zonula occludens-1 in respiratory and vascular endothelial cells [52,57], potentially facilitating translocation of MNPs across tissue barriers and exacerbating systemic exposure. Similarly, MNP-induced epithelial-to-mesenchymal transition in lung epithelial cells [53] could contribute to tissue fibrosis and chronic respiratory disease.

Fourth, endoplasmic reticulum stress and dysregulated autophagy were identified as additional mechanisms of MNP-induced cellular injury [43,45,47]. ER stress, triggered by accumulation of misfolded proteins, activates the unfolded protein response and, if unresolved, can lead to apoptosis. Autophagy, a cellular process responsible for degrading and recycling damaged organelles and proteins, was shown to be both activated and impaired by MNPs depending on exposure conditions, suggesting complex and context-dependent effects on cellular homeostasis.

Finally, the potential for MNPs to act as vectors for co-contaminants, including chemical additives (phthalates, bisphenols, and flame retardants) and adsorbed environmental pollutants (heavy metals, persistent organic pollutants), adds an additional layer of toxicological complexity [15]. These substances can leach from MNPs upon ingestion or inhalation, compounding the direct toxic effects of the particles themselves and potentially acting through endocrine-disrupting and carcinogenic pathways. For example, plastic additives such as bisphenol A and certain phthalates are well-established endocrine disruptors [60], while adsorbed organic pollutants alongside MNPs may alter biological processes, thereby disrupting endocrine and immune systems in humans [61]. The dynamic interactions between particle characteristics, sorbed contaminants, and host biological systems complicate efforts to disentangle particle-specific effects from chemical co-exposure effects.

### 4.3. Public Health and Clinical Implications

The findings of this review have significant implications for public health policy, clinical practice, and environmental health research. From a public health perspective, the ubiquity of MNP exposure and the mounting evidence of adverse health effects underscore the need for comprehensive strategies to reduce plastic pollution and human exposure. Policy interventions should prioritize upstream approaches, such as reducing single-use plastic production, improving waste management infrastructure, and promoting the development of biodegradable alternatives. Downstream interventions should focus on exposure reduction, particularly among high-risk populations, including advising consumers to limit use of bottled water and single-use plastic containers, which were identified as significant sources of MNP exposure in several studies [34,38,39,40]. Occupational health regulations should be strengthened to protect workers in plastic manufacturing, recycling, and textile industries, where exposure levels are likely to be substantially elevated [19].

From a clinical standpoint, healthcare providers should be aware of MNPs as a potential environmental risk factor contributing to chronic disease burden. Although causal relationships have not yet been definitively established, the associations documented in this review, particularly the links between MNP exposure and cardiovascular events, gastrointestinal inflammation, respiratory disease, and reproductive health outcomes, suggest that MNPs may be a relevant consideration in clinical assessments of patients with unexplained or treatment-refractory conditions. Future research should explore whether biomonitoring of MNP exposure could be integrated into clinical risk assessment tools, particularly for vulnerable populations such as pregnant women, individuals with pre-existing inflammatory or cardiovascular conditions, and those with high occupational or lifestyle-related exposures.

### 4.4. Methodological Considerations and Future Research Directions

This review identified several methodological limitations that must be addressed to advance the field. First, the lack of standardized methods for MNP detection, quantification, and characterization across studies limits comparability and hinders the establishment of consensus exposure thresholds. Studies employed diverse analytical techniques, including pyrolysis-gas chromatography/mass spectrometry (Py-GC/MS), laser direct infrared spectroscopy (LDIR), Raman spectroscopy, and scanning electron microscopy (SEM), each with distinct sensitivities, detection limits, and biases. Such methodological diversity introduces potential measurement error and exposure misclassification, which may partially explain discrepancies in reported concentrations and effect estimates across studies. As a result, direct quantitative comparison of exposure levels or pooled risk estimation remains challenging. Given the diverse analytical techniques employed across studies, there is urgent need to establish standardized reporting frameworks, minimum quality control criteria, and harmonized exposure protocols (e.g., certified reference materials), essential to enhance comparability, enable meta-analytic synthesis, improve the interpretability of emerging epidemiologic evidence, and reproducibility.

Second, the geographic distribution of the included studies was heavily skewed, with approximately 60% of studies (*n* = 17) conducted in China. This concentration may limit the global generalizability of the findings [62], as exposure sources, including plastic waste management practices, dietary habits, and environmental regulations, can differ substantially across regions. In many Western countries, established waste collection systems, higher recycling rates, more robust wastewater treatment, and environmental regulations can reduce the environmental burden of mismanaged plastics, potentially limiting downstream contamination of air, water, and food chains [63]. For example, in Canada, established waste collection and recycling infrastructure has enabled measurable diversion of plastic waste into material recovery systems, with over one quarter (27%) of discarded plastic being diverted for recycling and reuse in 2021, demonstrating the presence of organized waste management systems that can help mitigate environmental contamination [64]. Conversely, in parts of Asia and Africa, rapid industrialization, combined with limited waste management capacity and the legal or illegal transboundary movement of plastic waste from Western regions, has been associated with higher volumes of mismanaged plastic entering aquatic and terrestrial environments, thereby elevating the potential for human exposure through contaminated food and water sources, including seafood [65,66,67]. For example, countries in Southeast Asia, including Malaysia, Indonesia, and the Philippines, have been identified among the highest consumers of microplastics (MPs) out of 109 evaluated nations, with estimated daily intake in Indonesia increasing approximately 59-fold between 1990 and 2018, according to a study by Cornell University [68].

These differences highlight that exposure routes and risks may not be uniform globally, emphasizing the need for regionally tailored studies that account for distinct environmental contexts and dietary practices when interpreting health outcomes. Future research should aim to include more diverse populations, and subgroup analyses comparing populations across different regions may help elucidate regional differences in exposure profiles and associated health outcomes. Expanding cohort studies to include diverse populations for different geographic regions will also enhance the external validity of MNP health impact assessments and better inform globally relevant public health strategies.

Third, most clinical studies included in this review employed cross-sectional or case–control designs with small sample sizes and convenience sampling, which limits causal inference and generalizability. To strengthen causal understanding, future research should prioritize large-scale, population-based cohort studies with repeated measurements of MNP exposure over time. Longitudinal assessment would allow researchers to track changes in exposure and the subsequent development of health outcomes, thereby establishing temporal relationships. Incorporating repeated biomonitoring, along with detailed characterization of exposure sources and concentrations, can help identify dose–response patterns and cumulative lifetime effects. Additionally, such studies could examine critical windows of susceptibility (e.g., prenatal, early childhood) and interactions with other environmental and genetic risk factors.

Fourth, the reliance on in vitro models, while valuable for elucidating mechanisms, does not fully capture the complexity of human physiology, including systemic circulation, organ interactions, immune responses, and metabolic processing of MNPs. Future mechanistic research should incorporate more sophisticated models, such as three-dimensional organoids, organ-on-a-chip systems, and ex vivo tissue cultures, to better simulate in vivo conditions. Additionally, animal models using physiologically relevant exposure routes and doses may help bridge the gap between in vitro findings and human health outcomes, though care must be taken to account for species differences in MNP toxicokinetics and toxicodynamics.

Fifth, dose–response relationships and exposure thresholds remain poorly defined. While in vitro studies consistently demonstrate concentration-dependent effects, the concentrations used often far exceeded those detected in human biological samples, raising questions about the translational relevance of observed toxicity to real-world exposure scenarios. For example, experimental studies frequently use milligram-per-milliliter (mg/mL) levels of MNPs, whereas human biomonitoring data indicate exposure in the nanogram to microgram-per-milliliter (ng–µg/mL) range [44,50,69]. Although high-dose experiments are valuable for elucidating mechanistic pathways and hazard potential, supraphysiologic exposure levels may exaggerate oxidative, inflammatory, and cytotoxic responses that do not fully reflect chronic, low-level exposures experienced by human populations. Future in vitro research should therefore prioritize environmentally relevant and biomonitoring-informed MNP concentrations, incorporate chronic low-dose and repeated exposure paradigms, and identify no-observed-adverse-effect levels (NOAELs) and benchmark doses to improve risk assessment accuracy and translational validity.

Sixth, individual variability in susceptibility to MNP toxicity is poorly understood. Factors such as age, sex, genetic polymorphisms (e.g., in antioxidant or xenobiotic metabolism genes), pre-existing health conditions, nutritional status, and concurrent environmental exposures may modify MNP health effects. For example, prenatal and early-life exposures represent critical windows of susceptibility, as developing fetuses are especially vulnerable to environmental contaminants due to immature biological barriers and rapid organogenesis, which can alter fetal development and increase the risk of adverse health outcomes [70,71]. Similarly, reproductive health may be differentially affected by MNP exposure; MPs have been detected in human placental and fetal tissues, and some evidence suggests associations between placental MP levels and adverse pregnancy outcomes such as reduced birthweight, low Apgar scores, and smaller head circumference in newborns [72]. In addition, emerging animal research indicates that sex may influence microplastic effects on cardiovascular outcomes, with male subjects showing greater susceptibility to microplastic-induced atherosclerotic changes than females, potentially due to hormonal or genetic differences [73]. Pre-existing conditions such as cardiovascular disease may further modify susceptibility, as areas with high microplastic pollution have been linked to increased cardiometabolic disease risk in human populations [74]. These variable responses highlight the importance of incorporating stratified analyses, biomarker-based approaches, and consideration of life stage, sex, and health status in future studies to identify vulnerable subpopulations and tailor exposure reduction strategies effectively.

Finally, the relative contributions of different MNP polymer types, sizes, shapes, and surface modifications to toxicity remain incompletely characterized. While this review found evidence that polystyrene and polyvinyl chloride MNPs were particularly toxic in- vitro, and that smaller particles exhibited greater cellular uptake and effects, more systematic comparisons across polymer types and particle characteristics are needed to inform targeted risk management strategies.

### 4.5. Strengths and Limitations

This systematic review has several notable strengths. First, it integrates evidence from both clinical studies involving adult human participants and in vitro studies using human-derived cell lines, allowing for a more comprehensive assessment of MNP exposure across human body systems. Second, the review focuses on the recent literature, capturing advances in analytical techniques, exposure characterization, and mechanistic understanding that were largely absent from earlier syntheses. Third, adherence to PRISMA guidelines, use of multiple databases, independent screening by multiple reviewers, and quality appraisal of included clinical studies enhance the rigor, transparency, and reproducibility of the review.

Despite these strengths, several limitations warrant consideration. First, many clinical studies relied on small sample sizes and convenience sampling, which may limit generalizability and increase susceptibility to selection bias. Second, geographic representation was uneven, with a large proportion of studies conducted in China, limiting global generalizability and underscoring the need for population-based studies in diverse settings [62]. Finally, as with any systematic review, the findings are subject to publication bias, as studies reporting significant associations or toxic effects may be more likely to be published [75].

## 5. Conclusions

This systematic review synthesizes recent evidence on the health impacts of MNPs across human body systems by integrating findings from clinical studies in adult populations and in vitro investigations using human-derived cell lines. Collectively, the evidence demonstrates that MNP exposure is widespread, biologically relevant, and capable of disrupting key cellular and physiological processes through size-, polymer-, and concentration-dependent mechanisms. Although important gaps remain, particularly regarding standardized exposure assessment, long-term health outcomes, and population-level risk, the findings underscore MNPs as a growing environmental health concern rather than inert byproducts of plastic degradation. Continued interdisciplinary research is critical to understand causal inference over time and identify susceptible populations. Ultimately, advancing understanding of MNP-related health effects is essential for informing epidemiologic research, guiding regulatory decision-making, and developing evidence-based public health strategies aimed at reducing plastic-related exposures and protecting human health across the life course.

## Figures and Tables

**Table 1:** Inclusion and exclusion criteria.

Inclusion Criteria	Exclusion Criteria
1. Study participants aged 18 years or older 2. Male and female gender 3. Articles published in the last five years (2020–2025) 4. Articles published in English 5. Both original and review articles 6. Research associated with the impact of MNPs contamination in at least one human organ system 7. Research describing the mechanism of MNPs in at least one human organ system 8. Research describing prevalence of MNPs contamination in humans	1. Studies published before year 2020 2. Reports, letters, commentaries, pre-prints, abstracts only 3. Articles not published in English

**Table 2:** Research thread for all databases.

Search Strategies	Number of Studies Available
(nanoplastics OR microplastics) AND (organ OR health OR system OR genetic) AND (mechanism OR complication OR contamination OR toxic)	Total results: 1859 Embase (*n* = 25), Environment Complete (*n* = 1090), MEDLINE (*n* = 57), Scopus (*n* = 687)
Total number of duplicates removed	400
Total number of studies excluded based on eligibility criteria after initial screening	1188
Total number of studies excluded based on eligibility criteria after full text screening	241
Total number of studies used for the review	30

**Table 3:** Impact of MNPs on human body systems: findings from clinical studies.

Author (Ref.)	Type of Study	Human Body System Impacted	Major Findings	Quality Appraisal (out of 15)	Risk of Bias Assessment (out of 9)	Country of Study
Wang et al., 2024 [28]	Cross-sectional	Cardiovascular	*n* = 30; higher MP concentrations were significantly associated with greater severity of ischemic stroke, myocardial infarction, and deep venous thrombosis, and elevated D-dimer levels, indicating increased thrombotic activity.	8	7	China
Yang et al., 2024 [29]	Case–Control	Cardiovascular	*n* = 101; patients with acute coronary syndrome showed increased microplastic levels, particularly those with acute myocardial infarction and those at intermediate to high risk of coronary artery disease. Higher microplastic levels were also significantly associated with elevated inflammatory cytokines (IL-6 and IL-12p70) and increased B lymphocyte and natural killer cell counts.	12	5	China
Yu et al., 2024 [30]	Case–Control	Cardiovascular	*n* = 30; significantly higher MP concentrations were observed in patients with extracranial artery stenosis (ECAS) compared with those without ECAS, with higher levels associated with greater stenosis severity. The ECAS group also exhibited elevated D-dimer levels and prolonged thrombin time relative to the normal group.	9	6	China
Yan et al., 2022 [31]	Case–Control	Gastrointestinal	*n* = 102; individuals with inflammatory bowel disease had significantly higher fecal MP concentrations (41.8 items/g dry mass) compared with healthy individuals (28.0 items/g dry mass).	12	6	China
Cetin et al., 2023 [32]	Case–Control	Gastrointestinal	*n* = 31; tumoral colon tissues contain higher levels of MP than non-tumoral colon tissues, suggesting a possible association between colorectal cancer and MP exposure. MP particle sizes in tumoral colon tissues range from 1 to 1299 µm and include polyethylene, poly(methyl methacrylate), and nylon (polyamide).	9	4	Switzerland
Wu et al. 2025 [33]	Cohort	Gastrointestinal	*n* = 10; high-risk practices, such as frequent invasive gastrointestinal examinations, increased microplastic accumulation in fibrotic intestines. MP concentrations were significantly higher at lesion sites compared with surrounding tissues. In more fibrotic regions of Crohn’s fibrosis and involved ileum, PU and AUR concentrations were elevated, while CPE and Fluororubber levels decreased.	10	3	China
Li et al., 2025 [34]	Case–Control	Musculoskeletal	*n* = 55; polyvinyl chloride and polyethylene terephthalate were the most prevalent MP polymers identified among participants, with notable exposure risks from sources such as bottled water and take-out containers. Microplastics also exhibited a significant toxic effect on osteoblasts, suggesting a potential correlation with the progression of osteoporosis.	11	4	China
Liu et al., 2025 [35]	Cross-sectional	Respiratory	*n* = 50; increased single-type and overall MP exposure was significantly associated with a higher risk of severe community-acquired pneumonia. MP concentrations in bronchoalveolar lavage fluid were also significantly correlated with changes in respiratory microbiota, including reduced α-diversity, and with multiple inflammatory factors.	10	7	China
Zhang et al., 2024 [36]	Cross-Sectional	Reproductive	*n* = 113; exposure to polytetrafluoroethylene (PTFE) was significantly associated with poorer semen quality, including reductions in total sperm count, sperm concentration, and progressive motility.	11	7	China
Kim et al., 2025 [37]	Cross-sectional	Reproductive	*n* = 13; in endometrial stromal cells, smaller plastic particles exhibited greater cellular uptake than larger particles, with significant morphological change and cell death occurring at concentrations above 100 µg/mL after 24 h. MPs and NPs accumulated in the cytoplasm and nuclei, with uptake rates dependent on particle size	6	4	South Korea
Xu et al., 2025 [38]	Case–Control	Reproductive	*n* = 45; MP burden was observed to rise with increasing severity of cervical cancer. Metabolomic profiling identified D-mannose and cis,cis-muconic acid as the metabolites demonstrating greatest differences. Pathway enrichment analysis highlighted amino sugar and nucleotide sugar metabolism as key pathways potentially linking MP exposure to cervical cancer progression.	10	4	China
Xu et al., 2025 [39]	Case–Control	Reproductive	*n* = 80; exposure levels of MP are significantly higher in tissues with uterine fibroids compared with normal tissues from healthy individuals. Exposure to PE-MP is also associated with an increased risk of uterine fibroids compared with healthy individuals. A positive correlation observed between MP exposure levels and uterine fibroid size.	11	4	China
Zhang et al., 2025 [40]	Case–Control	Reproductive	*n* = 45; individuals with pregnancy-induced hypertension (PIH) exhibited significantly elevated levels of PE and polycarbonate in umbilical cord samples compared to controls. Overall MP concentrations were approximately 1.46-fold greater among PIH cases. Although the use of plastic tableware, seafood consumption, and intake of beverages packaged in plastic were identified as possible contributing factors, these variables were not significant after multivariable adjustment. MP presence was positively associated with the use of plastic food containers and consumption of takeout meals and was further correlated with poorer neonatal outcomes, including reduced Apgar scores and higher neonatal mortality rates.	10	4	China

Adverse impact of MNPs on human body systems; AUR—acrylate copolymer; CPE—chlorinated polyethylene; ECAS—extracranial artery stenosis; IL—interleukin; MP—microplastic; MNP—microplastic and nanoplastic; NP—nanoplastic; PE—polyethylene; PIH—pregnancy-induced hypertension; PS—polystyrene; PU—polyurethane.

**Table 4:** Impact of MNPs on human body systems: findings from in vitro studies.

Author (Ref.)	Human Body System Impacted	Major Findings	Country of Study
Ma et al., 2025 [41]	Cardiovascular	At 0.1 µg/L, both 0.05 µm and 1 µm MNP particles suppressed myocyte contractility, decreased Ca^2+^ transient amplitude, and disrupted contraction and calcium (Ca^2+^) transient dynamics. In hypertrophic iPSC cardiomyocytes, 0.05 µm particles further exacerbated hypertrophy, evidenced by increased cell size and proBNP expression. Cardiotoxic effects were associated with mitochondrial dysfunction, including reduced mitochondrial membrane potential and increased mitochondrial and intracellular reactive oxygen species (ROS).	United States
Persiani et al., 2025 [42]	Cardiovascular	MPs, particularly PE and PS, impaired vascular smooth muscle cell viability, induced apoptosis, and triggered pathological alterations, including disrupted migration and proliferation, thereby increasing the risk of cardiovascular diseases such as atherosclerosis and vascular calcification.	Italy
Xue et al., 2025 [43]	Cardiovascular	In human cardiomyocytes AC16 cells, differentially expressed genes induced by PS-MP were predominantly enriched in pathways related to endoplasmic reticulum (ER) stress and autophagy, indicating activation of ER stress responses.	China
Chen et al., 2024 [44]	Gastrointestinal	After 48 h of exposure, PS-MPs entered into normal human liver (THLE-2) cells without inducing evident acute cytotoxicity at concentrations <20 µg/mL. Long-term exposure (90 days) to an environmentally relevant dose (0.2 µg/mL) significantly disrupted cellular metabolic profiles, with more pronounced effects observed for nanosized particles.	China
Guanglin & Shuqin, 2024 [45]	Gastrointestinal	Exposure to PS-NPs was found to disrupt iron homeostasis in esophageal cells and impair mitochondrial autophagy. These alterations contributed to increased mitochondrial ROS production, heightened inflammatory signaling, and increased cellular injury and death.	China
Nissen et al., 2024 [46]	Gastrointestinal	MP exposure caused an overgrowth of opportunistic bacterial groups, including *Enterobacteriaceae*, *Desulfovibrio* spp., *Clostridium* group I, and the *Atopobium*–*Collinsella* group, while concurrently reducing the abundance of beneficial taxa, except Lactobacillales.	Italy
Najahi et al., 2025 [47]	Gastrointestinal	Exposure to MPs of different sizes (1 µm and 2.6 µm) for 72 h caused significant decrease in cell viability, apoptosis, increased ROS production, and autophagy in Caco-2 cells.	Tunisia
Çobanoğlu et al., 2021 [48]	Lymphatic	Exposure significantly increased micronucleation, nucleoplasmic bridge formation, and nuclear bud formation in human peripheral blood lymphocytes.	Turkey
Weber et al., 2022 [49]	Immune	Exposure to NP caused primary human monocytes and monocyte-derived dendritic cells to secrete cytokines as key initiators of inflammation.	Germany
Koner et al., 2023 [50]	Immune	Exposure to PS-NPs (50–500 µg/mL) significantly reduced the viability of human macrophages. At 500 µg/mL, PS-NPs induced oxidative stress and decreased cell proliferation. PS-NP exposure also reduced mitochondrial membrane potential and caused DNA damage in macrophages.	India
Zhang et al., 2025 [51]	Reproductive	Exposure to PS-NPs for 48 h did not cause significant changes in cytotoxicity, Calcein intensity, or active mitochondrial levels in KGN human ovarian granulosa cells. However, PS-NP exposure resulted in a dose-dependent increase in cytoplasmic vacuolization, increased total lysosomal area, and a higher number of lipid droplets in KGN cells.	China
Dong et al., 2020 [52]	Respiratory	PS-MPs cause cytotoxic and inflammatory responses in BEAS-2B cells through reactive oxygen species (ROS) formation, resulting in reduced transepithelial electrical resistance and increasing the risk of chronic obstructive pulmonary disease.	Taiwan
Halimu et al., 2022 [53]	Respiratory	Exposure of Human alveoli epithelial A549 cells to PS-NP increased cell migration and epithelial–mesenchymal transition marker expression, alongside upregulation of ROS and NADPH oxidase 4. PS-NPs also induced mitochondrial dysfunction and endoplasmic reticulum stress.	China
Annangi et al., 2023 [54]	Respiratory	Human primary nasal epithelial cells exposure to polyethylene terephthalate NP caused an increase in intracellular ROS, LC3-II protein expression levels, expression of p62, and loss of mitochondrial membrane potential.	Spain
Han et al., 2024 [55]	Respiratory	Cellular uptake of PS-NPs in lung epithelial cells occurs primarily through an integrin α5β1-dependent endocytic pathway. Increased expression of integrin α5β1 amplified PS-NP internalization and intensified mitochondrial Ca^2+^ imbalance and depolarization. These mitochondrial disturbances promoted excessive ROS generation, inflammatory signaling, genomic damage, and necrotic cell death, thereby contributing to the development of pulmonary pathology.	China
Winiarska et al., 2024 [56]	Respiratory	NPs measuring 25 and 50 nm penetrated bronchial smooth muscle and small airway epithelial cells, impairing bioenergetics and inducing mitochondrial dysfunction compared to cells not treated with NPs.	Poland
Chen et al., 2023 [57]	Vascular	Exposure to PS-MP caused oxidative stress in human vascular endothelial EA. hy926 cells by reducing the expression of antioxidants, leading to apoptotic cytotoxicity. PS-MPs also induced vascular barrier dysfunction via the depletion of zonula occludens-1 protein.	Taiwan

Adverse impact of MNPs on human body systems; AUR—acrylate copolymer; CPE—chlorinated polyethylene; ECAS—extracranial artery stenosis; ER—endoplasmic reticulum; IL—interleukin; KGN cells—human ovarian granulosa cell line; MP—microplastic; MNP—microplastic and nanoplastic; NP—nanoplastic; PE—polyethylene; PIH—pregnancy-induced hypertension; PS—polystyrene; PU—polyurethane; ROS—reactive oxygen species; THLE-2 cells—normal human liver epithelial cells; 1uality appraisal was not conducted for in vitro studies, as these investigations used human-derived cell line.

**Table 5:** Micro- and nanoplastic (MNP) detection and characterization methods: typical detection limits, polymer specificity, and known biases/limitations.

Method	Detection Limit	Polymer Specificity	Main Strengths	Biases and Limitations
Optical Microscopy (light/stereo)	~100 µm	None-visual size/shape only	Simple, low cost	Misses <100 µm, cannot identify polymer type
Fluorescence Microscopy (Nile Red)	~1–50 µm	Greater visibility of hydrophobic particles	Rapid screening	Stains non-plastic organics, false positives
Fourier Transform Infrared (FTIR) Spectroscopy	~10–20 µm	High infrared (IR) spectral libraries	Polymer identification and size	Time-consuming and sensitive sample prep, limited to <10 µm
Raman Spectroscopy (µ-Raman)	~1 µm	High	Polymer identification and size	Fluorescence interference, time-consuming
Pyrolysis-GC/MS	Qualitative/ quantitative by mass	High	Identification and quantification by chem signature	Destroys morphology, time consuming
Thermal Desorption GC/MS (TD-GC/MS)	~ng mass	High	Minimal preparation	Cannot identify size or count particles
Scan/Trans Electron Microscopy (SEM/TEM) + En Dispers X-ray (EDX)	<100 nm imaging	Low–moderate (via EDX)	Morphology at nanoscale	Unreliable for low atomic number polymers (plastics), complex prep
Atomic Force Microscopy (AFM)-IR/nano-FTIR	~10–100 nm	Occasionally high	Nano-chemical mapping	Emerging technique, costly
Dynamic Light Scattering (DLS)	~1 nm	None	Rapid size distribution in suspension	Unreliable for large, mixed-sized samples
Nanoparticle Tracking Analysis (NTA)	~10–50 nm	None	Counts + size distribution	Cannot make chemical identification, RI-sensitive
Mass Spectrometry (ToF-SIMS)	<100 nm surface analysis	High (surface chem)	Detailed surface chemistry	Costly, generating complex data
Flow Cytometry (with fluorescent staining)	~200 nm–microns	Low	Counts in liquid suspension	Stain biasness, unreliable detection of polymer types

## Data Availability

No new data were created or analyzed in this study. Data sharing is not applicable to this article.

## Appendix A

**NEWCASTLE—OTTAWA QUALITY ASSESSMENT SCALE**
**CASE CONTROL STUDIES**
Note: A study can be awarded a maximum of one star for each numbered item within the Selection and Exposure categories. A maximum of two stars can be given for Comparability.
**Selection**
(1) Is the case definition adequate? (a) yes, with independent validation (b) yes, e.g., record linkage or based on self reports (c) no description
(2) Representativeness of the cases (a) consecutive or obviously representative series of cases (b) potential for selection biases or not stated
(3) Selection of Controls (a) community controls (b) hospital controls (c) no description
(4) Definition of Controls (a) no history of disease (endpoint) (b) no description of source
**Comparability**
(1) Comparability of cases and controls on the basis of the design or analysis (a) study controls for _______________ (Select the most important factor.) (b) study controls for any additional factor (These criteria could be modified to indicate specific control for a second important factor.)
**Exposure**
(1) Ascertainment of exposure (a) secure record (e.g., surgical records) (b) structured interview where blind to case/control status (c) interview not blinded to case/control status (d) written self report or medical record only (e) no description
(2) Same method of ascertainment for cases and controls (a) yes (b) no
(3) Non-Response rate (a) same rate for both groups (b) non respondents described (c) rate different and no designation

**NEWCASTLE—OTTAWA QUALITY ASSESSMENT SCALE**
**COHORT STUDIES**
Note: A study can be awarded a maximum of one star for each numbered item within the Selection and Outcome categories. A maximum of two stars can be given for Comparability.
**Selection**
(1) Representativeness of the exposed cohort (a) truly representative of the average _______________ (describe) in the community (b) somewhat representative of the average ______________ in the community (c) selected group of users, e.g., nurses, volunteers (d) no description of the derivation of the cohort
(2) Selection of the non-exposed cohort (a) drawn from the same community as the exposed cohort (b) drawn from a different source (c) no description of the derivation of the non-exposed cohort
(3) Ascertainment of exposure (a) secure record (e.g., surgical records) (b) structured interview (c) written self report (d) no description
(4) Demonstration that outcome of interest was not present at start of study (a) yes (b) no
**Comparability**
(1) Comparability of cohorts on the basis of the design or analysis (a) study controls for _____________ (select the most important factor) (b) study controls for any additional factor (These criteria could be modified to indicate specific control for a second important factor.)
**Outcome**
(1) Assessment of outcome (a) independent blind assessment (b) record linkage (c) self-report (d) no description
(2) Was follow-up long enough for outcomes to occur (a) yes (select an adequate follow up period for outcome of interest) (b) no
(3) Adequacy of follow up of cohorts (a) complete follow up—all subjects accounted for (b) subjects lost to follow up unlikely to introduce bias—small number lost—> ____ % (select an adequate %) follow up, or description provided of those lost) (c) follow up rate < ____% (select an adequate %) and no description of those lost (d) no statement

## Appendix B

**Table A1:** NOS-xs: adaptation of the NOS for cross-sectional studies—association studies.

Domain	Item	Answer (Only 1 per Item Possible)	Rating	Awarded Star(s)
STUDY SAMPLE SELECTION (max. 2 stars)	1. Representativeness of the study sample	(a) Truly representative of the target population (all subjects or random sampling).	✵	____/1 star
		(b) Somewhat representative of the target population (non-random sampling).	✵	
		(c) No representative of the target population (selected group of users).	***-***	
		(d) No description of the sampling strategy.	***-***	
	2. Sample size	(a) Justified and satisfactory.	✵	____/1 star
		(b) Not justified or unsatisfactory.	***-***	
ASSESSMENT of EXPOSURE and OUTCOME (max. 4 stars)	3. Assessment of the exposure(s)	(a) Gold-standard assessment tool.	✵✵	____/2 stars
		(b) Acceptable assessment tool.	✵	
		(c) Non-acceptable assessment methods.	***-***	
		(d) No description of the assessment tool.	**-**	
	4. Assessment of the outcome(s)	(a) Gold-standard assessment tool.	✵✵	____/2 stars
		(b) Acceptable assessment tool.	✵	
		(c) Non-acceptable assessment methods.	***-***	
		(d) No description of the measurement tool.	**-**	
CONFOUNDING FACTORS (max. 3 stars)	5. Adjustment for confounde(s)	(a) The study estimates are adjusted for all the most important confounding factors, and not adjusted for potential mediator(s).	✵✵	____/2 stars
		(b) The study estimates are adjusted for all the most important confounding factors, but also for potential mediator(s).	✵	
		(c) The study estimates are adjusted for some but not all the most important confounding factors.	✵	
		(d) The study estimates are not adjusted for any of the most important confounding factors or no description is provided.	**-**	
	6. Assessment of confounder(s)	(a) Most of the confounding factors controlled for were measured through gold standard assessment methods.	✵	____/1 star
		(b) Most of the confounding factors controlled for were measured through acceptable assessment methods.	✵	
		(c) Most of the confounding factors controlled for were not measured through at least acceptable assessment methods.	**-**	
		(d) No confounding factors controlled for.	**-**	
**Total score**				____**/9 stars**

Adapted from Carra et al. 2025 [24]. Number of stars are awarded per domain indicate the weighting of individual items (for example, two starts indicate more weight than one star). Three differently colored bocks were used for each of the three domains.
